# The Association Between Work Ability Support Services and Work Disability With the Mainly Used Health Care Sector in Mental Health Disorders and Musculoskeletal Diseases

**DOI:** 10.1097/JOM.0000000000003758

**Published:** 2026-05-18

**Authors:** Anna Siukola, Mikko Korhonen, Lauri Vähätalo, Elina Kervinen, Tiia Reho, Klas Winell, Mervi Viljamaa, Riitta Sauni

**Affiliations:** 1 Faculty of Medicine and Health Technology, Tampere University, Tampere, Finland (A.S., M.K., L.V., E.K., T.R., R.S.); 2 Conmedic Oy, Espoo, Finland (K.W.); 3 Pihlajalinna Lääkärikeskukset Oy, Tampere, Finland (M.V.).

**Keywords:** musculoskeletal diseases, mental disorders, occupational health care services, work ability support

## Abstract

**Objective:**

This study examined the association between the health care sector mainly used, the use of work ability support services, and work disability among occupational health care (OHC) patients with mental health disorders and musculoskeletal diseases—2 major contributors to work disability.

**Methods:**

Data from a nationwide OHC provider included sickness absences, disability benefits, health care contacts, and sociodemographics of 54,933 patients. Participants were grouped by diagnosis (F, M, F M) and categorized by their mainly used health care sector. Logistic regression analyzed associations.

**Results:**

Most patients mainly used OHC. Those relying on other health care sectors were less likely to receive work ability support and had higher odds of work disability.

**Conclusions:**

OHC should identify patients mainly using other sectors to ensure they receive necessary work ability support services.

## Abstract 2

Patients with mental health or musculoskeletal disorders who primarily use other health care sectors instead of occupational health are less likely to receive work ability support and more likely to become work disabled. Improved coordination and implementation of work ability services across all care sectors, especially occupational health, are essential.

## Graphical abstract

**Figure:**
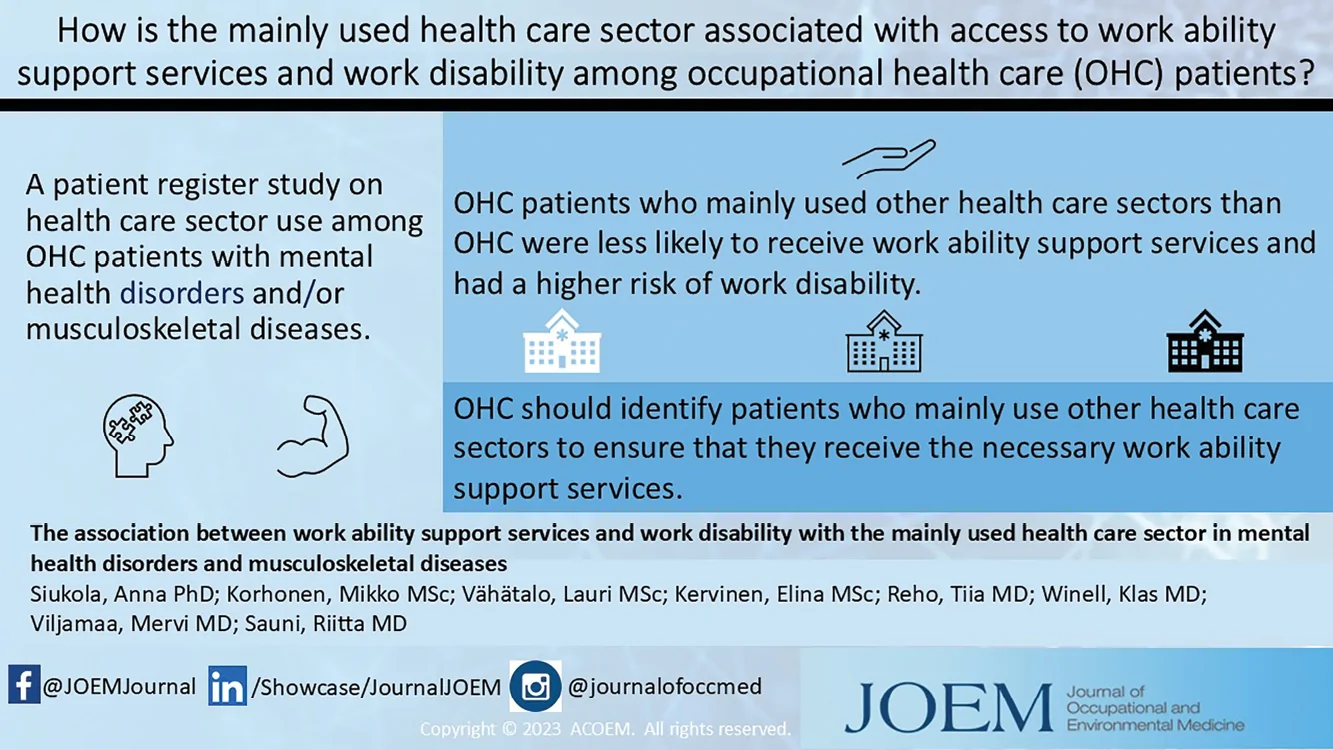

**LEARNING OUTCOMES**

- Users of occupational health care services also utilize other health care sectors in parallel, but it is not known how this relates to the implementation of work ability support services or to work disability.
- Among patients with mental health disorders and musculoskeletal diseases who mainly used health care sectors other than occupational health care to manage their illness, there was a lower likelihood of implementing work ability negotiations and work ability support, and a higher likelihood of work disability.
- In occupational health care, it is important to ensure that necessary work ability support services are implemented also for patients who use other health care sectors.

In a situation of labor shortage, it is essential to secure the workforce’s work ability.^1^ Musculoskeletal diseases and mental health disorders are the primary causes of long-term sickness absences and disability pensions among the working-age population in many countries.^2–4^ In granted sickness allowances, mental health disorders have surpassed musculoskeletal diseases in prevalence over the past 5 years. Mental health disorders and musculoskeletal diseases each account for approximately one-third of work disability cases.^3,4^ Mental health disorders and musculoskeletal diseases significantly increase the risk of work disability, and their co-occurrence multiplies this risk.^5^ Thus, this study focuses on these 2 diagnostic groups.

Finnish occupational health care (OHC) has 2 simultaneous roles: mandatory preventive functions and voluntary primary care (curative) functions. OHC primary care operates as a parallel service to public and private care and does not restrict the use of these 2 services.^6^ Approximately 90% of employees in Finland are covered by OHC services. Of these, 94% have access to curative care services in addition to statutory preventive services.^7^ Work ability support is available to all employees covered by OHC, regardless of whether curative care is included in the agreement between the employer and the OHC provider.^8^ The employer’s statutory OHC includes workplace surveys, health examinations, provision of information, advice and guidance, monitoring of coping at work, referrals to rehabilitation, participation in the organization of first aid, planning of measures to maintain and promote work ability, and monitoring and evaluation of activities.^8^ It is voluntary to include curative care in OHC agreements, which means that the content of agreements and the coverage of OHC services vary greatly. Although work ability support is part of the employer’s statutory preventive services, in practice the provided curative care can support employees’ work ability. It has been stated that if workplaces have different capacities to provide work ability support, this may lead to a divergence in employees’ work ability at the population level.^9^

Employees who mainly use health care sectors other than OHC should also have the possibility to use work ability support services when needed. Work ability support services include work ability negotiations and the planning of adjustments to work tasks, flexible working hours, improvements to physical ergonomics, applying for partial sickness allowance, vocational and medical rehabilitation, or, as a last resort, applying for a disability pension.^9,10^ Work ability support also includes return-to-work support, the assessment of remaining work ability, and the evaluation of work disability. Even curative care visits may contain elements that promote work ability support.^11^ In this study, work ability support refers to work ability negotiations and the above-mentioned work ability support contacts implemented within OHC.

Although it is known that working-age employees in Finland mainly use OHC services,^12^ they also utilize other health care sectors. According to a register-based study, 10% of OHC patients used secondary care, 11% public primary health care, and 13% private health care in parallel with OHC.^13^ Based on a survey targeted at OHC patients, 85% had visited an OHC physician and 25% a public health center physician during the previous year.^14^ Another study reported that 52% of employees used only OHC services.^15^ In Finland, working-age individuals with low socioeconomic status, regardless of employment, mainly use public health care services.^16^ Among OHC patients, the largest user groups are manual workers and lower nonmanual employees.^17^ These same groups were also emphasized in studies examining patients who frequently use OHC services.^18^ Previous research has shown that employer-provided curative care increases OHC physician visits but decreases visits to public health care physicians.^19^ Conversely, higher socioeconomic status has been associated with an increased likelihood of using OHC or a combination of OHC and private health care services.^16^ However, differences in health service utilization (outpatient and hospital services; public, private, and OHC) between occupational groups cannot be fully explained by employees’ health status or sociodemographic factors.^20^

The health care sector utilized may have implications for an individual’s work ability. It has been observed that employees who used services from other health care sectors parallel with OHC had an increased risk of prolonged sickness absence and disability pension.^13^ It is not known, however, how well work ability support services are implemented in OHC in such situations. Although work ability support is part of preventive OHC, it is possible that the need for support is not identified or that support services are not actively offered if the treatment and follow-up occur outside OHC.

Work ability support services have been shown to have positive effects on work ability,^9^ making it necessary to examine whether these services are implemented differently among those who mainly use other health care sectors than OHC and whether the use of these services needs to be strengthened among those not mainly using OHC for curative care. There is a recognized need for more research on work ability support services implemented in OHC,^9,21^ which this study aims to address. To increase the knowledge related to disability prevention, this study examines how people with musculoskeletal diseases, mental health disorders, or both utilize different health care sectors and whether this has implications for the provision of work ability support. Additionally, we examine whether patients who mainly use OHC differ from those who mainly use other health care sectors in terms of the implementation of work ability support services and the outcome of work disability.

## METHODS

### Study Population and Data Formation

The study data consist of individuals who had used the services of a nationwide OHC provider at least twice between 2018 and 2022. OHC patient registry data were linked with the following registry data from the same period: disability pensions from the Finnish Centre for Pensions (ETK), sickness absences exceeding 10 days from the Social Insurance Institution of Finland (Kela), secondary care visit data from the Finnish Institute for Health and Welfare (THL) Care Register for Health Care (Hilmo), primary care visit data (health centers and private health care providers) from the THL’s Avohilmo, and sociodemographic data from Statistics Finland’s FOLK database. The OHC patient registry included information on curative care and work ability support contacts, as well as sickness absence data including also the first day of absence.

The study included individuals whose physician contact diagnosis during the study period was classified under ICD-10 as mental and behavioral disorders (F), musculoskeletal diseases (M), or both (FM) based on the OHC patient registry, Avohilmo or Hilmo (n = 109,511). Individuals with at least 1 F diagnosis but no M diagnosis were assigned to the F group, and those with at least 1 M diagnosis but no F diagnosis were assigned to the M group. Individuals with both F and M diagnoses formed the FM group.

The final study population included individuals who, during the period from January 1, 2019, to June 30, 2022, had a physician contact or sickness absence recorded in the OHC patient registry with an F and M diagnosis, or a sickness absence recorded in Kela’s registry with an F and M diagnosis. Each diagnosis had to be preceded by a 12-month period without any curative care contact or sickness absence for the same diagnostic group, and without any work ability support services (work ability negotiation or work ability support contact) either in-person or remotely. These criteria aimed to identify cases representing a new illness episode. The follow-up period was defined so that each individual had at least 180 days of follow-up, with the maximum follow-up being 730 days. After applying these criteria, the final sample size was n = 54,933 (Fig. 1).

**FIGURE 1.**
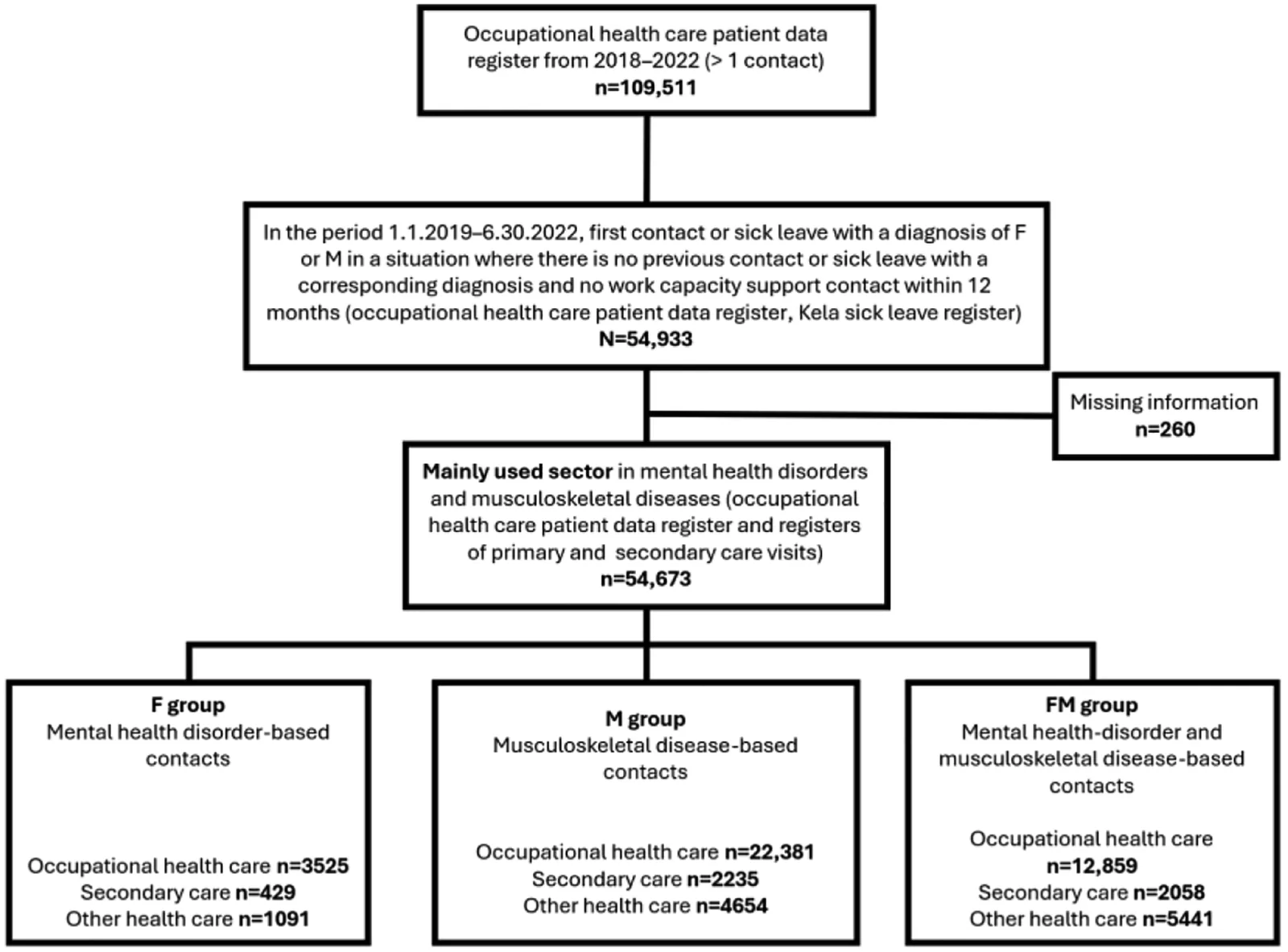
Study population.

In this study, the health care sectors considered were OHC, secondary care, and other health care. The category “other health care” included contacts in private and public primary health care. The mainly used health care sector utilized was defined based on the sector where each individual within the diagnostic group had the highest number of physician or nurse contacts related to the disease group from the start of the follow-up period. In other words, contacts in the F group included ICD-10 codes starting with F and ICPC-2 codes starting with P; contacts in the M group included ICD-10 codes starting with M and ICPC-2 codes starting with L; and the FM group included all of the above. After applying this definition and excluding missing data, the study population consisted of 54,673 individuals (Fig. 1). Based on the studied OHC patient registry, more than 95% of the participants had access to curative care services in addition to statutory OHC services.

### Variable Formation

Work disability as the outcome was defined as either receiving a disability pension decision or accumulating at least 300 sickness absence days within 2 years from the first F or M diagnosis. Disability pension decisions were identified from the Finnish Centre for Pensions registry and included the disability pension, the partial disability pension, the cash rehabilitation benefit, and the partial cash rehabilitation benefit (Finnish Centre for Pensions 2025). The calculation of sickness absence days was based on OHC patient records, supplemented with data from the Social Insurance Institution of Finland sickness allowance registry.

Work ability support services were identified from OHC patient records and included work ability support contacts, such as guidance and counseling provided by physicians, nurses, physiotherapists, psychologists, or social work professionals, as well as work ability assessments, for example, the evaluation of rehabilitation needs and the follow-up of rehabilitation. Work ability negotiations were examined as a separate work ability support measure. These preventive services are available to all employees, even if the treatment of illness occurs elsewhere.

### Statistical Analysis

The distribution of the background variables, including age, gender, and occupational group, was analyzed. The occupational group was categorized into the following groups: manual, lower nonmanual, upper nonmanual, entrepreneurial, and other professions. In addition, the frequencies of work ability support contacts, work ability negotiations, contacts in the mainly utilized health care sector, and sickness absences were analyzed. Furthermore, the association between the mainly utilized sector and work ability support services and work disability was examined. Analyses were conducted using logistic regression, with 3 models used for the association between the mainly utilized sector and work ability support: unadjusted, adjusted for gender and age, and adjusted for gender, age, and sickness absence days. For the association between the mainly utilized sector and work disability, adjustments were made for gender, age, and contacts related to curative care and work ability support. The reference group in all analyses consisted of individuals who mainly used OHC. Statistical significance was assessed at *P* < 0.05. Analyses were performed in Statistics Finland’s Fiona remote access environment using R version 4.2.2. The STROBE checklist is attached in the Supplementary Digital Content, https://links.lww.com/JOM/C513.

## RESULTS

The proportion of women in the study population (n = 54,673) was 64.0% in the F group, 48.5% in the M group, and 64.6% in the FM group (Table 1). The majority of those included in the F group were under 45 years of age, whereas in the M group most patients were over 45 years. In the FM group, the ages were distributed evenly around 45 years. In the F and FM groups, the largest occupational group was lower-non manual, while in the M group it was manual workers. When examining the use of work ability support services, it was observed that 39.9% in the F group, 26.8% in the M group, and 33.0% in the FM group had at least 1 work ability support contact within 90 days of the start of follow-up. During the same follow-up period, 5.6% of the F group, 0.9% of the M group, and 3.1% of the FM group participated in a work ability negotiation. Sickness absences were most frequent among those in the FM group and least frequent among those in the M group (Table 1).

**TABLE 1.** Background Information on Groups With F*, M†, and FM‡ Diagnoses (n = 54,673), and Their Distribution According to the Mainly Used Health Care Sector§

	F (n = 5045)	M (n = 29,270)	FM (n = 20,358)
	n (%)	n (%)	n (%)
Gender			
Female	3231 (64.0)	14,186 (48.5)	13,154 (64.6)
Male	1814 (36.0)	15,084 (51.5)	7204 (35.4)
Age group			
≤35	1821 (36.1)	4186 (14.3)	4764 (23.4)
36–45	1374 (27.2)	5907 (20.2)	5357 (26.3)
46–55	1082 (21.4)	8088 (27.6)	5346 (26.3)
56–65	710 (14.1)	9969 (34.1)	4592 (22.6)
>65	58 (1.1)	1120 (3.8)	299 (1.5)
Occupational group			
Manual	926 (18.4)	10,337 (35.4)	5335 (26.2)
Lower nonmanual	2190 (43.5)	9921 (34.0)	8831 (43.4)
Upper nonmanual	1119 (22.2)	5127 (17.6)	3378 (16.6)
Entrepreneurial	118 (2.3)	921 (3.2)	430 (2.1)
Other professions	678 (13.5)	2866 (9.8)	2358 (11.6)
Work ability support contacts			
At least 1 contact within 90 days from diagnosis	2012 (39.9)	7831 (26.8)	6722 (33.0)
At least 1 contact within 180 days from diagnosis	2262 (44.8)	9667 (33.0)	8450 (41.5)
Work ability negotiations			
At least 1 work ability negotiation within 90 days from diagnosis	280 (5.6)	273 (0.9)	631 (3.1)
At least 1 work ability negotiation within 180 days from diagnosis	387 (7.7)	514 (1.8)	990 (4.9)
Sickness absence days	Median/mean	Median/mean	Median/mean
Within a follow-up period of 180–730 days *median/mean* (days)	32/ 73	15/ 44	39/ 77
Mainly used sectors of health care services*	n (%)	n (%)	n (%)
Occupational health care	3525 (69.9)	22,381 (76.5)	12,859 (63.2)
Secondary care	429 (8.5)	2235 (7.6)	2058 (10.1)
Other health care¶	1091 (21.6)	4654 (15.9)	5441 (26.7)

* Mental health disorders.

† Musculoskeletal diseases.

‡ Mental health disorders and musculoskeletal diseases.

§ The primary service sector used was determined for the F group based on contacts coded with ICD-10 category F and ICPC-2 reason for encounter P, and for the M group based on ICD-10 category M and ICPC-2 reason for encounter L, after the first diagnosis, provided that there was a preceding 12-month period without sickness absence or contact within the respective diagnostic group and without any work ability support measures.

¶ Other health care included contacts in private and public primary health care.

The majority of the study population mainly used OHC services (Table 1). In the F group, less than one-tenth had mainly used secondary care, and slightly more than one-fifth had used other health care services mainly. In the M group, nearly one-fourth, and in the FM group, more than one-third had mainly used services other than OHC (Table 1).

When examining the number of sickness absence days in relation to the mainly utilized health care sector, the highest number of sickness absence days was observed among those who mainly used secondary care and the lowest among those who mainly used OHC (Table 2). Regardless of the mainly utilized sector, individuals in the M group had fewer sickness absence days compared with those in the F or FM groups (Table 2).

**TABLE 2.** Number of Individuals With Sickness Absence (n) and Their Average Number of Sickness Absence Days (Mean, Standard Deviation, Median) by Main Health Care Sector*, Grouped by Diagnosis Category (ICD-10: Mental Health Disorders F and Musculoskeletal Diseases M; n = 54,673)

Diagnosis Group	Mainly Used Health Care Sector									
	OHC			Secondary Care					Other Health Care†	
	Number of Individuals With Sickness Absence and Average Number of Sickness Absence Days									
	n	Mean (SD)	Median	n	Mean (SD)	Median	n	Mean (standard deviation)		Median
F	3525	53.6 (85.2)	25	429	160.0 (166.2)	97	1091	102.0 (120.3)		53
M	22,381	35.4 (70.7)	12	2235	98.0 (124.4)	53	4654	61.3 (99.2)		24
FM	12,859	62.3 (85.3)	32	2058	143.0 (151.5)	84	5441	88.2 (109.9)		46

* The main health care sector was defined as the sector with the most contacts during the follow-up period (minimum 180 days, maximum 730 days) after the first diagnosis. For the F group, contacts were identified by ICD-10 code F and ICPC-2 code P; for the M group by ICD-10 code M and ICPC-2 code L. This classification required a preceding 12-month period without sickness absence, contacts in the respective diagnosis group, or work ability support measures.

† Other health care included contacts in private and public primary health care.

Work ability negotiations were most common among those whose health care contacts mainly occurred in secondary care (15.6% in the F group, 7.2% in the M group, and 14.6% in the FM group; Table 3). Work ability support contacts were most frequent in all diagnostic groups among those who mainly used OHC (65.0% in the F group, 54.2% in the M group, and 73.0% in the FM group; Table 3). Among those who mainly used secondary care, 55.7% had work ability support contacts, compared with 43.8% for those who mainly used other health care services.

The likelihood of both work ability support contacts and work ability negotiations was lower among individuals who mainly used other sectors than OHC when the results were adjusted for age, gender, and sickness absence (Model 3; Table 3). When sickness absence was not included in the adjustment, those who mainly used secondary care had an increased likelihood of work ability negotiations, regardless of diagnostic group, compared with those who mainly used OHC. Individuals who mainly used other sectors than OHC had a lower likelihood of work ability support contacts across all diagnostic groups, regardless of whether the adjustments were applied or not (Table 3).

**TABLE 3.** Number and Percentage of Patients With a Work Ability Negotiation or a Work Ability Support Contact During 180–730 Days After First Diagnosis in Each Group, and the Association (OR) Between These Contacts and the Mainly Used Health Care Sector in the F, M, and FM Diagnosis Groups (n = 54,673)

Mainly Used Health Care Sector	Diagnosis Groups											
	F				M				FM			
		Model 1	Model 2*	Model 3†		Model 1	Model 2*	Model 3†		Model 1	Model 2*	Model 3†
	n (%)	OR (95% Cl)	OR (95% Cl)	OR (95% Cl)	n (%)	OR (95% Cl)	OR (95% Cl)	OR (95% Cl)	n (%)	OR (95% Cl)	OR (95% Cl)	OR (95% Cl)
Work ability negotiations (≥1)												
Occupational health care	441 (12.5)	ref	ref	ref	1027 (4.6)	ref	ref	ref	1616 (12.6)	ref	ref	ref
Other health care‡	120 (11.0)	0.86 (0.70–1.07)	0.888 (0.72–1.10)	**0.42 (0.33–0.55**)	229 (4.9)	1.08 (0.93–1.25)	1.06 (0.91–1.23)	**0.68 (0.57–0.80**)	534 (9.8)	**0.76 (0.68–0.84**)	**0.77 (0.70–0.86**)	**0.53 (0.47–0.60**)
Secondary care	67 (15.6)	1.29 (0.98–1.71)	**1.33 (1.01–1.77**)	**0.33 (0.23–0.48**)	161 (7.2)	**1.61 (1.36–1.92**)	**1.48 (1.24–1.76**	**0.54 (0.43–0.6**)	300 (14.6)	**1.19 (1.04–1.36**)	**1.208 (1.06–1.38**)	**0.46 (0.39–0.55**)
Work ability support contacts (≥1)												
Occupational health care	2291 (65.0)	ref	ref	ref	12123 (54.2)	ref	ref	ref	9393 (73.0)	ref	ref	ref
Other health care‡	478 (43.8)	**0.42 (0.37–0.48**)	**0.41 (0.36–0.47**)	**0.31 (0.27–0.36**)	2115 (45.4)	**0.71 (0.66–0.75**)	**0.69 (0.65–0.74**)	**0.58 (0.55–0.62**)	2812 (51.7)	**0.40 (0.37–0.42**)	**0.39 (0.36–0.41**)	**0.35 (0.33–0.37**)
Secondary care	239 (55.7)	**0.68 (0.55–0.83**)	**0.67 (0.54–0.82**)	**0.41 (0.33–0.51**)	1110 (49.7)	**0.84 (0.77–0.91**)	**0.79 (0.72–0.86**)	**0.54 (0.49–0.59**)	1201 (58.4)	**0.52 (0.47–0.57**)	**0.51 (0.46–0.56**)	**0.38 (0.34–0.42**)

* Adjusted for gender and age.

† Adjusted for gender, age, and sickness absence days.

‡ Other health care included contacts in private and public primary health care.

Individuals who mainly used a health care sector other than OHC had a higher likelihood of work disability compared with those who mainly used OHC (Table 4). Among patients in the FM group, the likelihood of work disability was elevated for those who mainly used secondary care (OR 7.55 [95% CI, 6.56–8.69]) and those who mainly used other health care services (OR 3.29 [95% CI, 2.91–3.72]) compared with those who mainly used OHC.

**TABLE 4.** Association Between the Mainly Used Health Care Sector and Risk of Work Disability (Granted Disability Pensions and ≥300 Sickness Absence Days Within 2 Years) in the F, M, and FM Diagnosis Groups, Adjusted for Gender, Age, and Health Care and Work Ability Support Contacts Accumulated 180–730 Days After the First Diagnosis

Mainly Used Health Care Sector	Diagnosis Groups					
	F		M		FM	
	n (%)	OR (95% Cl)	n (%)	OR (95% Cl)	n (%)	OR (95% Cl)
Occupational health care	124 (33.2)	ref	822 (54.8)	ref	778 (40.0)	ref
Other health care*	142 (38.1)	**6.75 (5.06–9.01**)	373 (24.9)	**2.92 (2.53–3.38**)	654 (33.7)	**3.29 (2.91–3.72**)
Secondary care	107 (28.7)	**12.84 (9.21–17.89**)	305 (20.3)	**4.64 (3.95–5.46**)	511 (26.3)	**7.55 (6.56–8.69**)
Total	373 (100)		1500 (100)		1943 (100)	

Bold values indicate statistically significant results (*P* < 0.05).

* Other health care included contacts in private and public primary health care.

## DISCUSSION

In the study population, OHC was the most commonly used health care sector. Those who mainly used it had more work ability support contacts than users of other sectors, while work ability negotiations were most frequent among patients mainly using secondary care. After adjusting for sickness absence, primary users of other sectors than OHC had clearly fewer work ability support contacts and negotiations across all study groups, and they faced a higher risk of work disability.

It is reasonable to assume that individuals who mainly use OHC have more work ability support contacts than those who mainly use other health care sectors. They may be more aware of work ability support services, and they may be guided to these services during other OHC contacts or may have better access to such services compared with patients who mainly use other sectors. Although work ability support services are available to all employees regardless of the type of OHC agreement,^8^ the implementation of work ability support ultimately depends on practices agreed upon between the employer and OHC service provider. Therefore, even though all individuals in this study population had access to work ability support as OHC patients, the content and intensity of support may have varied considerably, even among those who mainly used OHC.

It is known that patients who exclusively use OHC services have a lower likelihood of receiving a disability pension compared with those who use OHC in addition to other health care sectors.^22^ Although this study did not monitor the volume of service use, the findings may support this observation, as individuals who mainly used a sector other than OHC were found to have an increased risk of work disability. This may indicate that the most severe cases are managed within secondary care or that the need for work ability support has not been adequately addressed in OHC.

An important question is how work ability support services provided by OHC could prevent work disability among patients who mainly receive care from other health care sectors. At a minimum, identifying patients who are mainly treated elsewhere would be essential for ensuring the implementation of work ability support. If it is known that a patient frequently uses services outside OHC, it is crucial for OHC to assess the impact of the illness on functional capacity and work ability, evaluate the need for work ability support, and initiate appropriate work ability support services.

Furthermore, it is worth considering how collaboration between health care sectors could be strengthened and care better coordinated so that individuals requiring work ability support are referred from other sectors to OHC, ensuring that support is available to all employed individuals who need it, regardless of the health care sector they mainly used.^22^ In Finland, positive results have been achieved through the TYÖOTE model in collaboration between secondary care and OHC, where secondary care issues only a short sickness absence certificate and refers the patient to OHC for return-to-work support.^23,24^ This model is currently being extended to primary health care.^25^ It can be assumed that such practices improve referral to work ability support services. Unfortunately, it was not possible to account for the implementation of this model within the study population.

Work ability negotiations and sickness absence days were most frequent among individuals who mainly used secondary care. The likelihood of work ability negotiations was higher in this group compared with those who mainly used OHC when analyses were adjusted for age and gender. However, when sickness absence was included as an adjustment factor, the likelihood was reversed, indicating that patients with mental health disorders or musculoskeletal diseases who mainly used secondary care had a lower likelihood of work ability negotiations compared with those who mainly used OHC. At the same time, many workplaces and OHC providers have systems to identify individuals with high level of sickness absence and direct various support services to these employees. If information on an employee’s sickness absence is conveyed from the employer to OHC service provider as intended, it is possible that OHC assesses the work ability of an employee who mainly use secondary care differently from those who mainly use OHC services. The OHC may infer that, because the patient is currently receiving treatment within secondary care, the primary responsibility for care remains with that sector. Alternatively, the OHC may assume that the intensity of the treatment or the complexity of the clinical issue renders work ability support premature until the secondary care episode has concluded. Overall, the results suggest that individuals with similar levels of sickness absence may have different opportunities to access or to be referred to work ability negotiations depending on the main health care sector used.

Previous research examining the reasons for sickness absence among working-age individuals who frequently use multiple health care sectors found that mental health disorders were more prevalent in public primary health care, while musculoskeletal diseases were more common in OHC.^18^ In the present study, both mental health disorders and musculoskeletal diseases were most often treated in OHC. For those covered by OHC, it is relevant to consider the role of work ability support services in supporting mental health. For example, employees may be less willing to seek help for mental health issues within OHC if they perceive it as being too closely connected to their employer.^26^ Addressing these perceptions may require examining how strategic work ability management^27^ could mitigate such barriers and foster trust in OHC services. It is already known that workplace practices for managing work disability and the role of supervisors are essential to improve job modification and efforts to return to work for workers with low back pain.^28^

One-fourth of individuals in this study were mainly treated in other health care or secondary care, which may reflect the transfer of multimorbid patients to primary health care. These individuals should be identified in OHC owing to their elevated risk of work disability^5^ and sickness absence, and their need for work ability support should be assessed. Effective implementation of this support has been shown to require the strengthening of mental health expertise in both occupational and primary health care.^29^ However, in this study, the FM group did not differ from other groups in terms of using work ability support services or work disability risk.

Work ability negotiations were examined as a separate work ability support measure because they are specific to OHC and are not provided by other health care sectors. These negotiations were more frequently implemented in mental health disorders than musculoskeletal diseases. Work ability negotiations play a key role in information exchange, interaction, and building trust between the employee, supervisor, and OHC professionals. This discussion provides an opportunity to plan adjustments that align work demands with the employee’s functional capacity, with the primary goal of supporting continued employment.^30^

The increasing rates of work disability due to mental health disorders have attracted attention at the societal level, as reflected in initiatives such as the National Mental Health Strategy 2020–2030^31^ and the Mental Health Toolkit for Workplaces^32^ in Finland. It is worth considering whether this heightened societal focus on mental health is reflected in the findings of this study, potentially resulting in more mental health disorder-than musculoskeletal disease-related visits to OHC.

Although musculoskeletal diseases may also require negotiations, for example, when planning work modifications, workplaces may initially have the capacity to implement ergonomic solutions independently. In cases where musculoskeletal diseases require surgical intervention, work ability negotiations are often involved, but they may be postponed until after the surgery. Concrete work arrangements agreed upon during work ability negotiations have been shown to improve work ability. For instance, changes to working hours and job tasks based on these negotiations have been associated with reduced sickness absence episodes during follow-up.^33^ Work modifications may vary depending on whether the underlying cause of work disability is a physical impairment^34^ or a mental health condition.^35^

Users of OHC services differ in their access to services depending on the scope of coverage provided by their employer.^36^ In this study, most participants had access to OHC services that included curative care. The occupational group distribution of the study population was similar to that in previous research,^17^ where the largest groups were manual workers and lower nonmanual employees. Thus, there is no indication that the study population differed from the general OHC patient profile or consisted primarily of individuals in higher socioeconomic positions, who are more likely to use OHC services.^16^

Geographical location of the workplace or the patient’s residence may also influence service utilization. A study examining public mental health services for working-age individuals found that in rural areas, the availability of mental health services was not associated with disability pensions, whereas in urban and semiurban areas, a connection was observed.^37^ Future research could explore whether the availability of local OHC services is associated with the use of public mental health services and how geographical location affects this relationship. Further analysis could examine whether differences in service provision across well-being service counties are linked to disability pension rates.

A strength of this study is the large OHC patient registry, based on nationwide data from a major service provider. The dataset was supplemented with other registry information, enabling a comprehensive analysis of explanatory factors. The study focused on diagnostic groups most commonly associated with sickness absence and disability pensions among working-age individuals in Finland—mental health disorders and musculoskeletal diseases—thus addressing a large and societally significant population. Additionally, the study contributes valuable knowledge, as research on work ability support services in OHC and their association with the mainly used health care sector has been relatively scarce. The study highlights the patient subgroup who mainly use other health care sectors. These patients’ opportunities to receive work ability support should receive greater attention in the future.

A limitation of the study is that mental health disorders and musculoskeletal diseases were examined at the broad ICD-10-chapter level, meaning that individuals in the F and M diagnostic groups had conditions of varying severity and scope. The comprehensiveness of OHC agreements was also unknown. Furthermore, the registry data did not provide information on whether the participants had continuous access to the services of the OHC provider throughout the study period. However, the results were verified using a model requiring at least 1 OHC contact every 6 months during follow-up, and the findings were consistent with those of the main study population. It is also known that documentation practices related to work ability support services vary among OHC physicians,^38^ which may orient our findings in 1 direction or the other. For these reasons, other OHC providers’ registers should be included in future studies to diminish the variation caused by company rules. If OHC interprets a visit related to work ability as preventive care rather than curative care at an early stage, this may emphasize the implementation of work ability support services and contribute to differences observed between sectors. A noticeable detail is also the COVID-19 pandemic, which changed health care provisioning drastically by emphasizing remote care. Unfortunately, based on the available data, it could not be determined how COVID-19 might have affected the results.

## CONCLUSIONS

The main health care sector utilized appears to be associated with the number of work ability support contacts among OHC patients seeking care for mental health disorders and musculoskeletal diseases. Work ability support contacts were most frequent among those who mainly used OHC. Patients who mainly used health care sectors other than OHC were found to have a higher likelihood of work disability. OHC should pay particular attention to patients who, due to an illness, use other health care services more frequently than OHC in order to assess their functional capacity in a timely manner and ensure adequate work ability support services. This requires collaboration between health care sectors, which has already been promoted through the TYÖOTE initiative, where patients in need of work ability support are referred from secondary care to OHC as quickly as possible. Furthermore, to improve equitable access to work ability support services, process refinement is needed. Information on the use of different sectors could in the future be obtained through more advanced information systems or via patient surveys or health examinations, with appropriate consideration of data protection issues.

## ACKNOWLEDGMENTS

We wish to thank Tampere University Language services for the language revision.

## Supplementary Material


